# A data set of symptoms and needs of individuals affected by COVID-19

**DOI:** 10.1038/s41597-024-02961-6

**Published:** 2024-01-24

**Authors:** H. B. Stanley, M. Bensafi

**Affiliations:** Université Claude Bernard Lyon 1, CNRS, INSERM, Centre de Recherche en Neurosciences de Lyon CRNL U1028 UMR5292, NEUROPOP, F-69500 Bron, France

**Keywords:** Quality of life, Human behaviour

## Abstract

Here we provide data from an online survey of 639 people diagnosed with COVID-19 and resident in France, who were diagnosed with COVID-19 between 30th Jan 2020 and 29th August 2022. In addition to demographic information the survey includes questions about participants’ symptoms (by category), symptom onset and persistence, and the effects these symptoms had on their daily lives. Participants were able to include information related to their perceived medical, social and professional needs. These data are needed in order to create effective care policies addressing post-COVID sequelae. Information related to symptom association & dynamics is expected to be useful to clinicians and may also inform more fundamental studies.

## Abstract (summary)

**Table:** 

Measurement(s)	Physical & Mental Health, Demographics, Perceptions & Needs
Technology Type	Questionnaire
Sample Characteristic - Organism	Adult Humans
Sample Characteristic - Environment	Online Survey
Sample Characteristic - Location	France

## Background & Summary

This study was designed to provide data for scientists, clinicians and policy makers in order to assist them in the creation of effective policies caring for those affected by COVID-19. Specifically, we aimed to define the resources needed (in terms of financial support, social services or medical care) to meet people’s needs, and to plan for future pandemics. Despite the fact that there have been hundreds of millions of cases of COVID-19 worldwide since the start of the pandemic^1^ we still lack relevant data regarding the long-term consequences of this illness and the impact that chronic problems have on people’s everyday lives. Nevertheless it is becoming clear that disabling long-term symptoms arise even for people with apparently mild initial illness^2,3^ and it is therefore urgent to implement effective mitigation and rehabilitation strategies to minimise the impact of these symptoms on affected individuals and on society as a whole. We have collected data characterizing the wide-ranging symptoms of COVID-19, together with their effects on quality of life and the perceived needs of those affected. These data document symptom associations and dynamics and characterise the ways in which symptoms handicap individuals in their everyday lives. It is possible to analyse these data by age and/or gender, specific symptom categories or groups of symptoms, and use them to determine individuals’ perceived medical, social and professional needs. We expect the wide-ranging overview of COVID-19 symptoms to be useful to both clinicians and to researchers investigating fundamental questions, and the objective *and subjective* information to inform public policies.

## Methods

### Procedure and inclusion

The dataset^4^ is from a large-scale survey based on a questionnaire designed to be anonymous with no collection of IP addresses, written in French and posted online on the website https://project.crnl.fr/covid/. The title and introduction to the survey explained that the purpose of the survey was to understand better the effects of the disease on people’s daily lives and needs. It was advertised via our scientific network, and people were also able to find it via internet searching. Adults, resident in France, with an official diagnosis of COVID were invited to complete the survey, which was approved by the Institutional Review Board of INSERM (IRB00003888, IORG0003254, FWA00005831) of the French Institute of medical research and health, under number 21–805. Participants were not remunerated and were not monitored. All participants provided informed consent.

Data were collected between 15th July 2021 and 6th September 2022. Exclusion criteria were: persons under the age of 18 years, those without a diagnosis of COVID-19 (made either via an analytical test or by a doctor on the basis of symptoms alone), those who failed to complete the entire survey, or were not filling it in for the first time and those not resident in France. We have also excluded participants with diagnosis dates prior to the first cases of COVID-19 in France (3 participants), participants entering a recovery date prior to their diagnosis date (13 participants), and participants who omitted to provide a diagnosis date (5 participants). Combined these criteria excluded 415 of the 1054 participants. We did not use any criteria with respect to specific symptoms, diagnosis dates (other than the implausible dates as stated above), interval since diagnosis, duration of illness or perceived recovery.

### Data collected

**Fig. 1:**
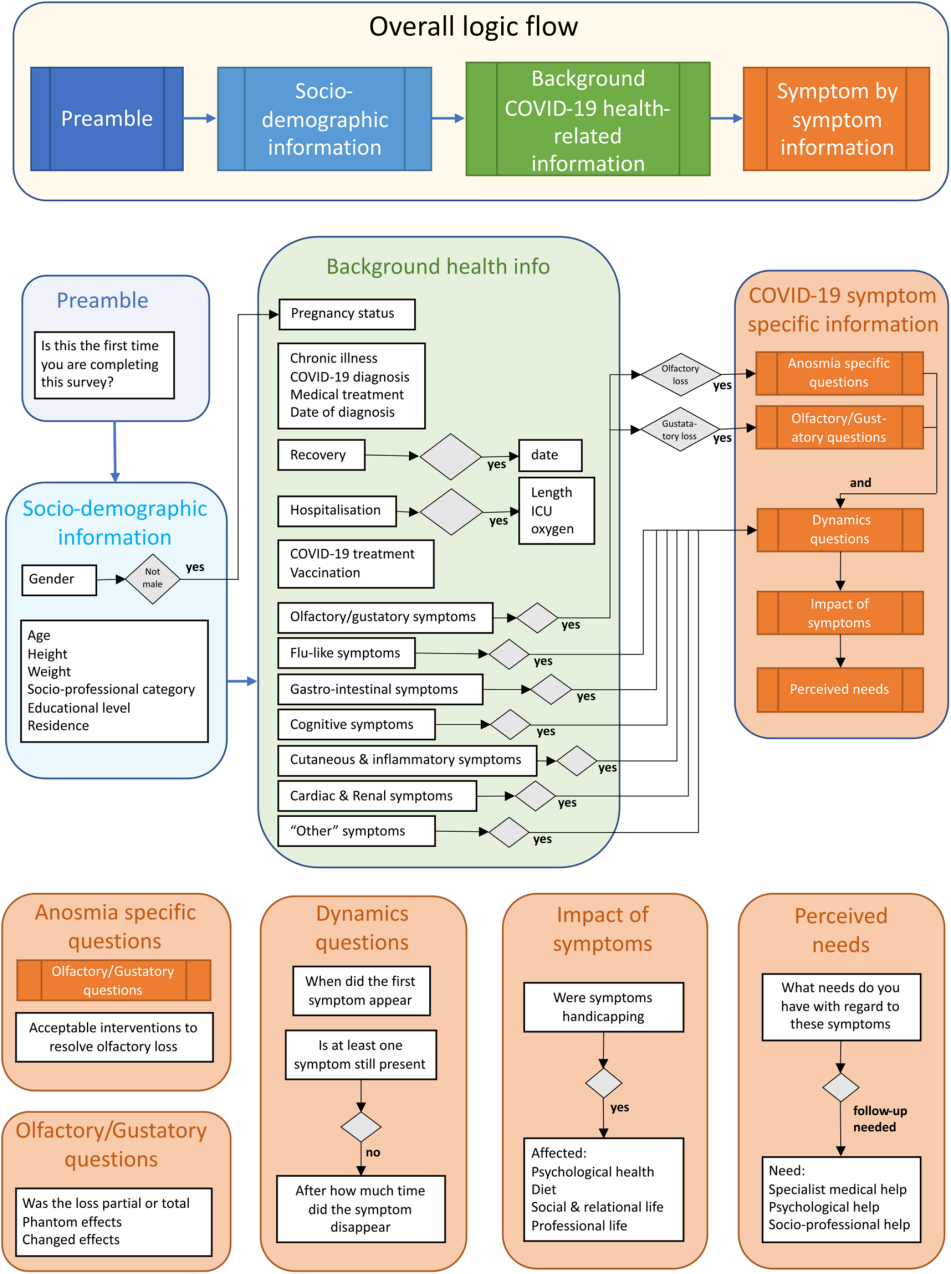
Overall structure of the questionnaire.

The questionnaire began with an explanation of the purpose of the study and consent. It continued with general compulsory questions and subsequently used a branching design. Figure 1 illustrates the structure of the questionnaire.

The first section of the questionnaire concerns demographic information: gender, age, height, weight, socio-professional category, education & profession, French department of residence and whether they lived in an urban, suburban or rural area. These questions were compulsory and there are no missing values.

General background COVID-19 and health information was then collected which included: their pregnancy status (if female), any chronic illnesses and regular medication, whether they are (or were) smokers, whether they had been vaccinated against COVID-19, and if so, with which vaccine.

There then followed a series of questions related to their illness with COVID-19. These data include how and when they were diagnosed with COVID-19, whether they were diagnosed with a specific variant, and if so which, whether they considered themselves recovered, and if so on what date, whether they were hospitalised, and if so whether they were given oxygen, and/or admitted to ICU, and what treatments (if any) they were given for their COVID-19 illness.

The following sections were specific questions relating to symptoms experienced by category. The categories were chosen with respect to WHO data available at the time of creating the survey namely: olfactory & gustatory symptoms (which included loss of smell and/or taste together with questions relating to changed or phantom smells and/or tastes); flu-like symptoms (fever, chills, dizziness, cough, sore throat, fatigue, shortness of breath, muscle weakness, muscle pain (aches) or joint pain, headache); gastro-intestinal symptoms (difficulty swallowing, difficulty eating/drinking, loss of appetite, nausea or vomiting, diarrhea); cognitive, neurological and psychiatric symptoms (migraines, memory problems, attention disorders, speech disorders, altered consciousness, confusion, serious neurological disorders, irritability, anxiety, depression, sleep disorders); cutaneous and inflammatory symptoms (rash, itching, hair loss, tooth loss, conjunctivitis); cardiac and renal symptoms (kidney problems, heart problems, chest pain) and finally they were asked about any other symptoms that had not already been covered. For each symptom category participants reported experiencing they were asked about symptom onset and whether or not the symptom was still present. If they had recovered they were asked after how long. They were systematically asked whether they found the symptom a handicap in their everyday lives and whether the symptom had affected their psychological health, diet, social & relational life and professional life. Finally, people were asked to describe their needs categorised as specialist medical, psychological/psychiatric and socio-professional. These questions were asked on a true/false basis, but participants were also able to provide verbatim free responses. Some additional questions were asked related to potential therapies people might accept to redress their olfactory and gustatory symptoms.

We also provide data related to the questionnaire completion itself, namely the date and time of submission, the time participants spent completing various sections of the questionnaire and the total time spent.

### Data pre-processing

LimeSurvey, a tool that creates online questionnaires and surveys, was used to collect the data. The raw data were first exported from LimeSurvey in xlsx format. Preprocessing was then performed to filter out incomplete or duplicate responses, respondents not residing in France, minors, and responses with inconsistent dates. Although the questionnaire was completely anonymous, participants were given the opportunity to describe symptoms, needs, or other information in dedicated boxes. To ensure the anonymity of the database, this information has not been included. The final filtered database was saved in csv format.

## Data Records

The dataset^4^ consists of a text readme file, a pdf with the survey questionnaire, three descriptive json files and a csv format database containing the 639 responses. In this data base each row corresponds to a single participant and each column to a variable. The descriptive json files (Table 1) put the study in context and provide a detailed description of the variables. The final json file lists the variables in the original French and in English translation. We also provide a copy of the questionnaire in pdf format.

**Table 1:** Description of files composing the dataset.

Filename	Description
0-Readme.txt	description of the files in the dataset
1-Covid-19 - Symptoms - Impact on quality of life and - needs of affected people - Raw data.csv	data
2-Covid-19 - Symptoms - Impact on quality of life and - needs of affected people - Descriptive_metadata.json	description of the data
3-Covid-19 - Symptoms - Impact on quality of life and - needs of affected people -data_configuration-french.json	data configuration (in French) defining variable names with conditional branches and possible responses
4-Covid-19 - Symptoms - Impact on quality of life and - needs of affected people -data_configuration-english translation	Database column numbers & original variables followed by data base column numbers & English translations
online_questionnaire_fr.pdf	printed version of the online questionnaire (in French)

## Technical Validation

We provide the following analyses to facilitate evaluation of the validity and limitations of the data.

### Diagnosis date

**Fig. 2:**
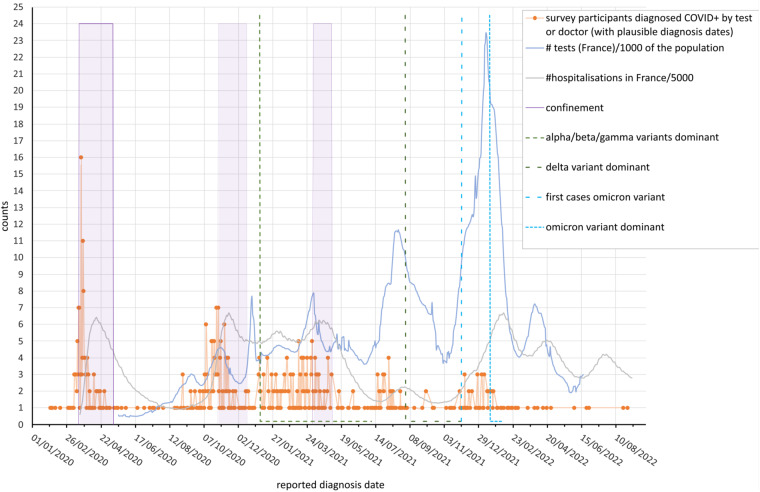
COVID pandemic in France compared with diagnosis dates of the 639 survey participants. Orange dots: Number of survey participants as a function of diagnosis date. Grey line: Number of hospitalizations in France/5000 of the population and blue line: Number of COVID-19 tests in France/1000 of the French population using data from^19^. Purple periods: Periods of confinement in France. Dotted lines indicate time periods where testing showed different dominant variants in metropolitan France (data from^10,18^).

Recorded diagnosis dates closely follow the known waves of infection in France^5^ (see Fig. 2).

### Correspondence between diagnosis date and Covid testing proportion

**Fig. 3:**
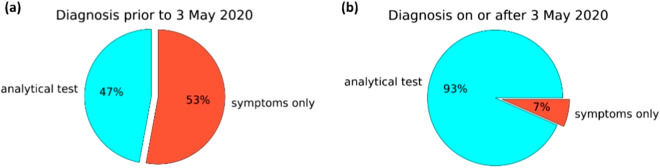
Proportion of participants diagnosed by test or doctor by date. Cyan: Participants diagnosed by analytical test; Red: participants diagnosed by a doctor on the basis of their symptoms alone (**a**) before 3rd May 2020 and (**b**) on or after 3rd May 2020.

The lock-down imposed in France lasted from 17th March 2020 to 3rd May 2020. During this time very few people were tested for COVID infection nationally. The data from our survey reflect this. Indeed, among the 138 participants diagnosed before 3/05/2020, 65 (47%) were diagnosed by analytical tests and 73 (53%) by a medical doctor. In contrast, among the 501 participants diagnosed on or after 3/05/2020, 468 (93%) were diagnosed by analytical test and only 33 (7%) by a doctor on the basis of symptoms alone (see Fig. 3).

### Geographical distribution

**Fig. 4:**
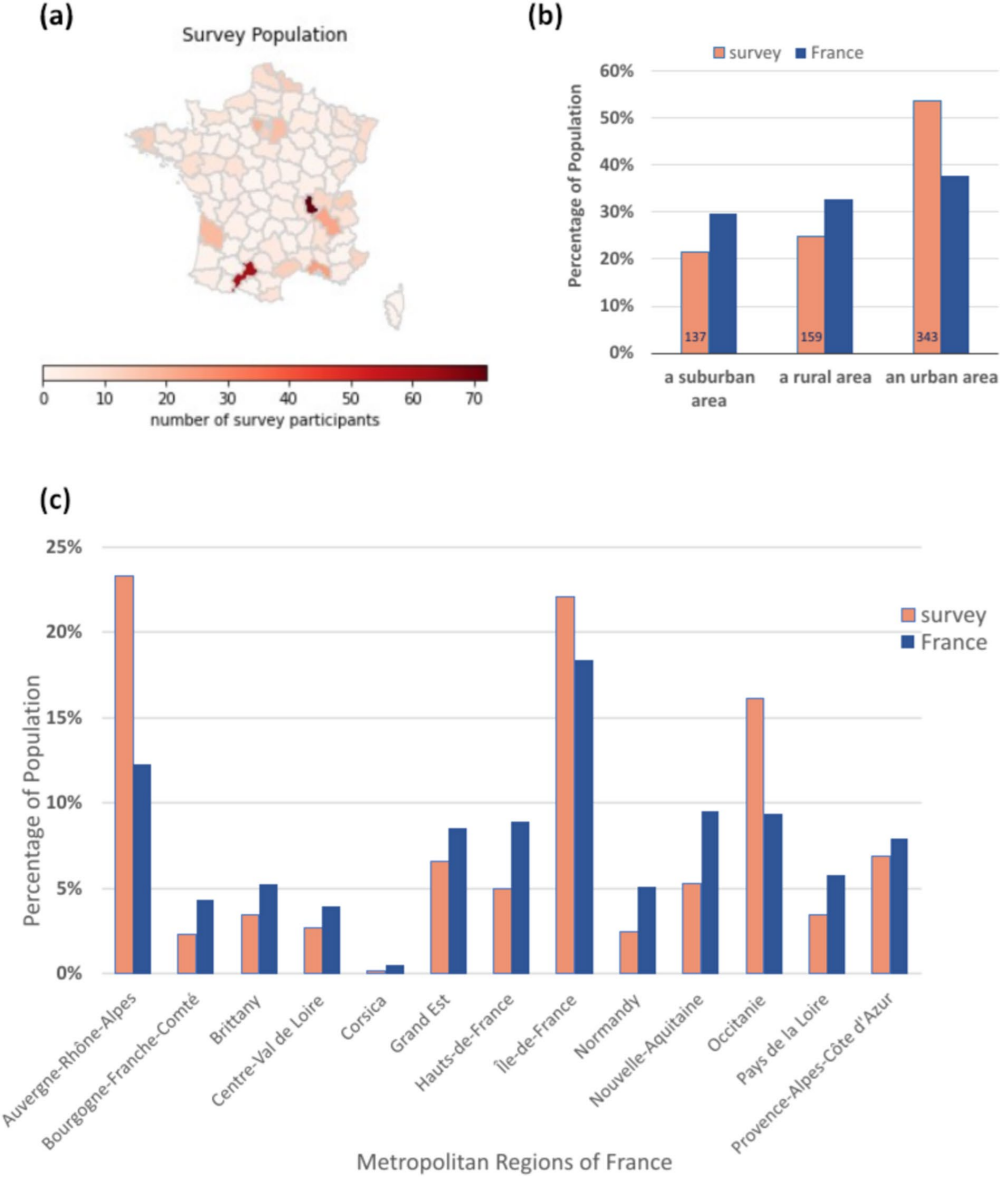
Residence. (**a**) geographical distribution of participants across metropolitan France, (**b**) comparison of the proportion of survey participants resident in suburban, rural and urban areas compared with the equivalent proportions in France, and (**c**) comparison of proportion of survey participants by French region with the proportion of the French population by region. French population data from^6^.

**Fig. 5:**
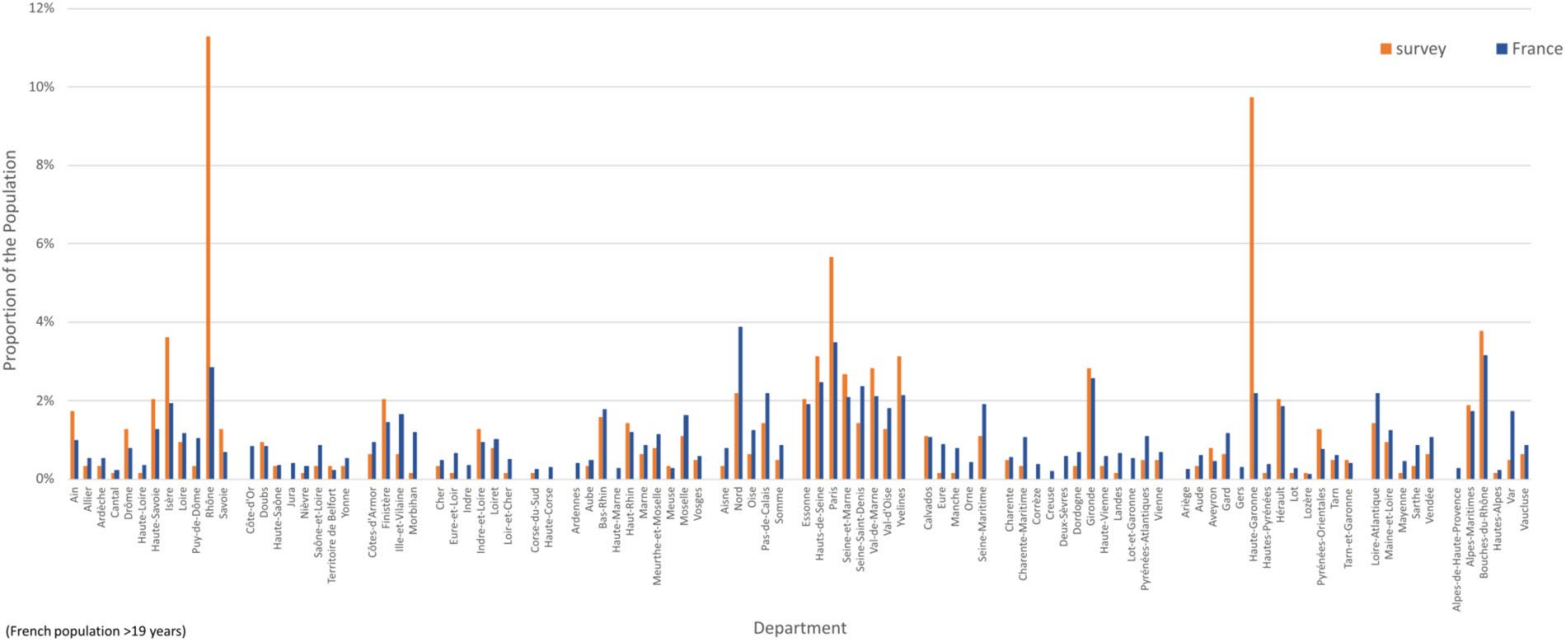
Comparison of proportion of survey population by French department, with the proportion of the French population (at least 20 years old) by department. Departments are grouped by region. The departments with greatest difference (Rhône and Haute-Garonne) correspond to the over-representation caused by our publicity in the Lyon and Toulouse areas, and lead to the corresponding over-representation of the populations of the regions Auvergne-Rhône-Alpes and Occitanie (data from^6^).

We have good geographical distribution of participants across metropolitan France (Fig. 4a) consistent with French government statistical data^6^ (Fig. 4c). The over-sampling of the Lyon, Toulouse & Paris areas reflects the impact of publicity from our academic partners (see Fig. 5). 137 people (21% of the survey population) live in a suburban area, 159 (25%) in a rural area and 343 (54%) in an urban area^7^. We have a small over sampling of the urban population, which is typical for online surveys (see Fig. 4b).

In Fig. 5 we provide a comparison between the proportion of survey participants by French department with the proportion on the general French population.

### Average age of hospitalized people

The average age of hospitalized people is greater than that of un-hospitalized people (average hospitalized 50.2 [95% CI 46.7,53.7] years compared with average age not hospitalized 42.5 [95% CI 41.5,43.6] years) p < 0.001, calculated using the software package jamovi^8^) and the average age of those who reported experiencing flu-like and at least one other symptom (43.8 [95% CI 42.8,44.8] years) is higher than the average age of those who reported experiencing flu-like symptoms only (39.7 [95% CI 36.9,42.6] years) (p = 0.003), which agrees with known information relating to the vulnerability of people to COVID-19 increasing with age.

### Prevalence of anosmia

We note that our data include participants diagnosed from the very beginning of the pandemic and also periods when the alpha, beta & gamma variants were circulating and the prevalence of anosmia may vary not only with variant but with vaccination status or other factors. Nevertheless, our data are consistent with the literature^9^. Only 7% of our survey participants (43) were diagnosed after 01/01/2022 when the omicron variant of COVID-19 became prevalent in France^10^ and the incidence of anosmia declined^11^. Excluding these participants, 61% of our participants reported olfactory loss which is comparable to the results of Menni *et al*.^11^ who quote a prevalence of 52.7% anosmia during the delta wave.

### Percentage of smokers

110 people (17%) were either occasional or regular smokers, which compares to 18.5% of people over 18 years old in France^12^.

### Preponderance of women

We note that online surveys typically have a preponderance of women^13–16^. Our population is 77% female which is comparable with the data of Ferdenzi *et al*.^14^ whose online survey of French COVID-19 sufferers was 78% female.

## Usage Notes

### Population studied

The survey focused on the symptoms and needs of patients who had a COVID-19 infection, regardless of any subjective persistence of symptoms. Information relates to individuals’ subjective perception of their health, with no independent verification either of the severity of the symptoms nor of their source. The data do not focus specifically on the so-called “long COVID-19” population (although many participants fall into this category).

**Fig. 6:**
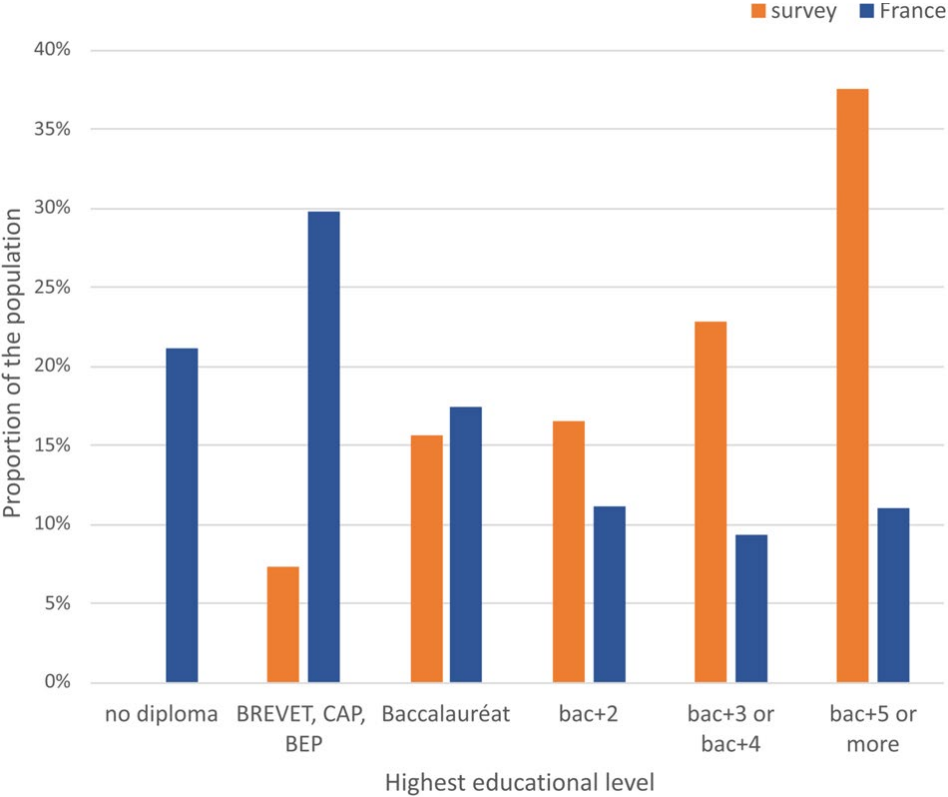
Comparison of the educational levels of the survey population compared with those of the general population of France (data from^20^). Bac + 2 means that the individual has successfully completed two years of higher education (Baccalauréat + 2 years).

**Fig. 7:**
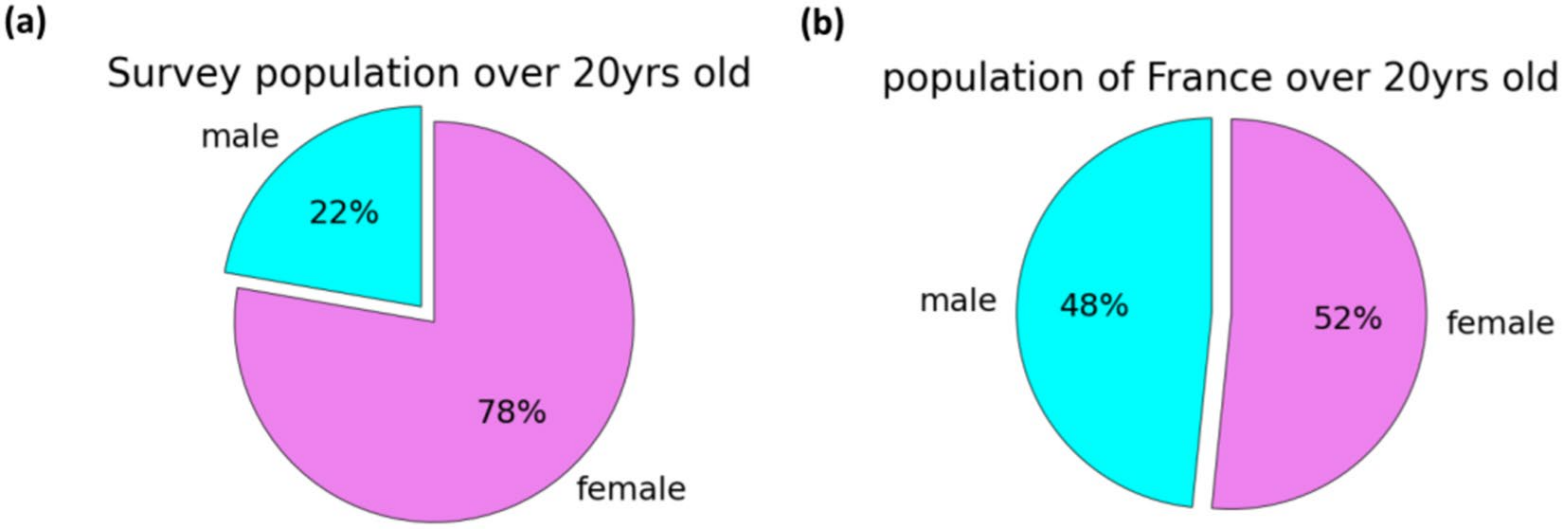
Comparison of the gender ratio of the survey population (**a**) with that of metropolitan France in 2021 (**b**) (data from^6^).

We are only able to include data from people who chose to complete the online survey. The data therefore suffer from selection biases (over representation of urban, educated individuals, women, and symptomatic individuals, and exclude those unable to access the internet) (see Figs. 6, 7).

#### Age distribution of the survey population

**Fig. 8:**
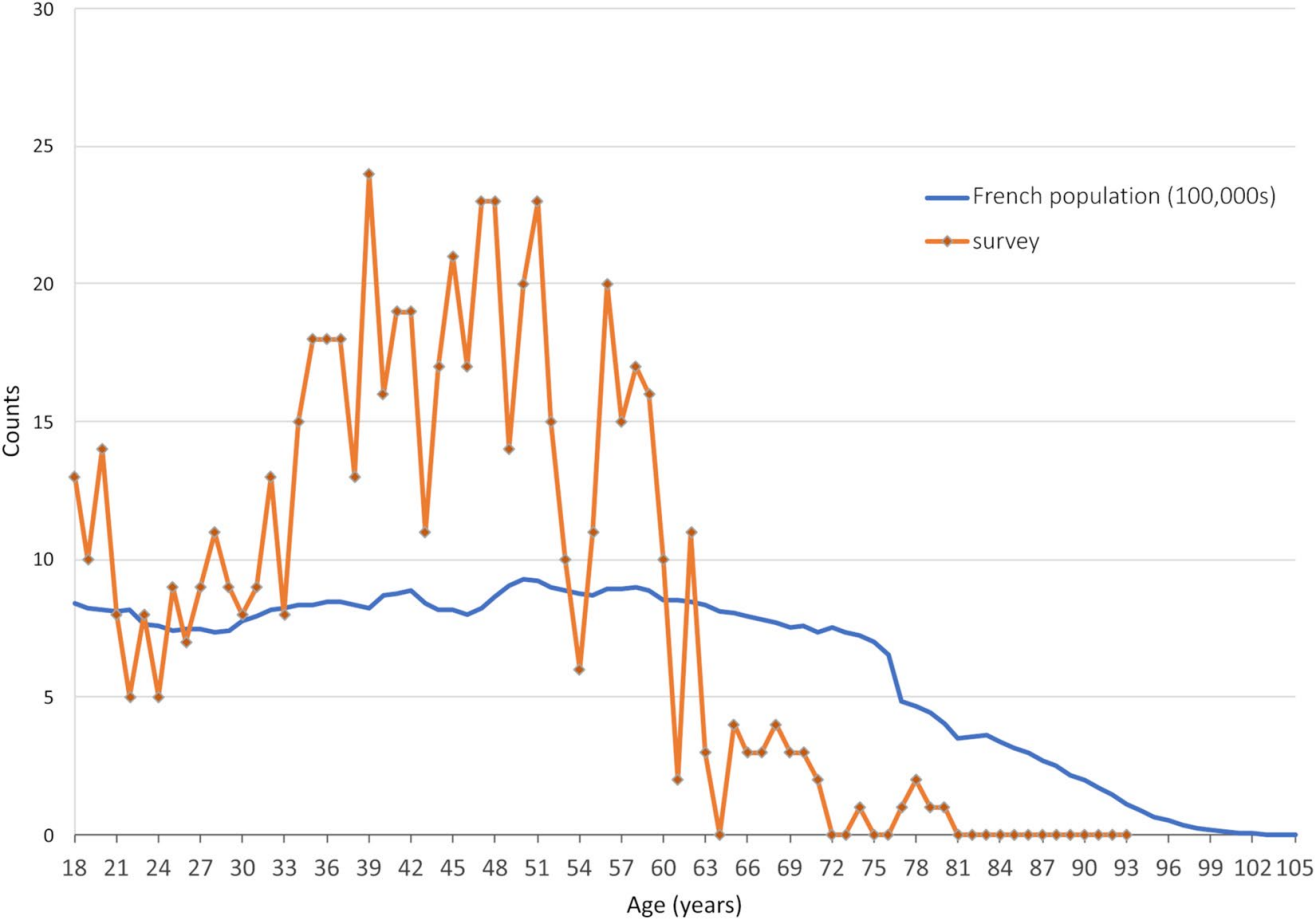
Comparison of the age distribution of the survey population compared with that of the general population of France 2023 (data from^17^). Only 24 survey participants are over 65 years old and 8 survey participants (1.3% of the survey population) are over 70 years old.

Children were excluded from the survey and, although the survey was open to the elderly, general inability to use the internet (poor eyesight, computer illiteracy, lack of access etc) means that the survey is heavily biased towards working age (under 65 year old) persons. In Fig. 8 we compare the age distribution of the survey population with that of France in 2023 (data from^17^).

#### Delay between infection and reporting

**Fig. 9:**
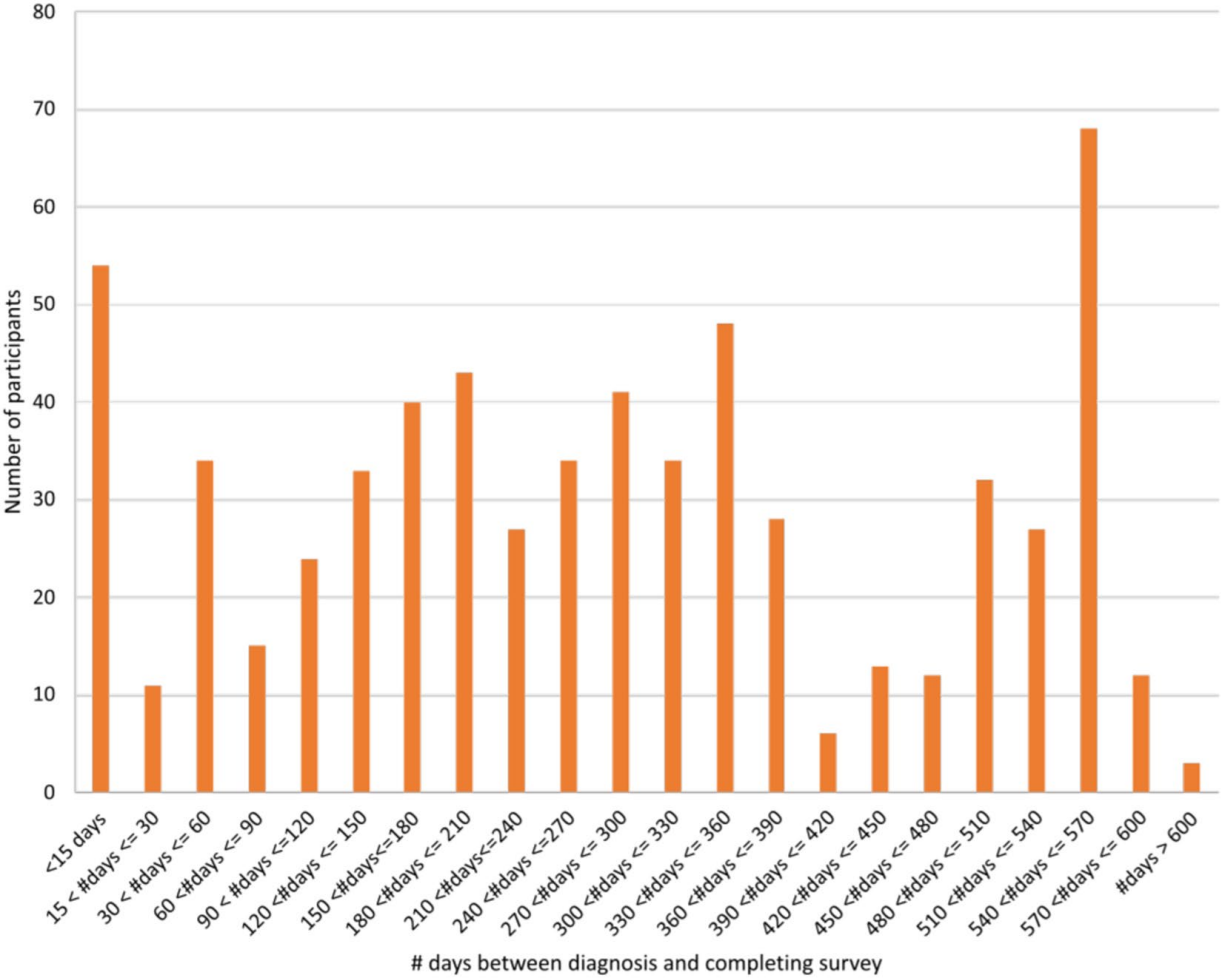
Delay between initial diagnosis of COVID-19 infection and completing the survey. 54 participants completed the survey within two weeks of infection and 192 more than a year after diagnosis.

This is variable between individuals (see Fig. 9). It is possible to filter data to focus on, or exclude, specific groups, based on the time elapsed between diagnosis and completing the survey.

#### Variants

The diagnosis dates cover waves of infection with different dominant variants, with an under-representation of the delta & omicron variants. We do not have sufficient confirmed diagnoses of the variant of infection (this was an open question) to enable a robust statistical analysis comparing the different variants. Although it is possible to match the diagnosis date to the predominance of variants at that time, we note that for the first six months of 2021 the alpha, beta and gamma variants circulated mostly concomitantly in France^18^.

#### Declared recovery

A significant proportion of participants declared themselves cured of COVID-19, yet later described a number of persistent symptoms (80/200). We consider these responses, which at first sight seem counterintuitive, to be linked to the fact that declaring oneself cured of COVID-19 depends in part on subjective factors, on the individual perception of each person. This individual perception, which our data show to be variable from one person to another, is possibly constructed on the basis of the appreciation of the severity of the persistent symptoms, or of the feeling that people have still not recovered their initial state of health. In fact, all of this suggests that there is no simple definition of who is considered cured or not cured.

## Data Availability

Data filtering and statistical analysis was performed using the open access software jamovi^8^. No custom code was used for the curation or validation of the dataset.
